# Comprehensive Unloading Strategy for Rapid Heart Recovery Under Support With Impella

**DOI:** 10.7759/cureus.45590

**Published:** 2023-09-20

**Authors:** Masayoshi Yamamoto, Tomoko Ishizu, Kentaro Minami, Ryosuke Tsuchiya, Daigo Hiraya

**Affiliations:** 1 Department of Cardiology, Faculty of Medicine, University of Tsukuba, Tsukuba, JPN

**Keywords:** heart failure, cardiogenic shock, unloading, ivabradine, impella

## Abstract

The establishment of a strategy for rapid heart recovery in patients with cardiogenic shock is required. Impella is a percutaneous left ventricular (LV) assist device that maintains hemodynamic stability and also causes LV mechanical unloading. However, the timing at which Impella should be started and a systematic strategy after the start of Impella have not been established. We report a representative case of dilated cardiomyopathy requiring catecholamines and intra-aortic balloon pumping (IABP). The hemodynamics were unstable under IABP support, and withdrawal from IABP or catecholamines was considered impossible. However, the exchange of the IABP with Impella CP made it possible to suppress the heart rate with ivabradine, introduce intensive heart failure medication, and discontinue catecholamines. The patient was weaned from Impella 24 days after the start of the first Impella CP. Rapid heart recovery was achieved with favorable outcomes. We present a comprehensive strategy for rapid heart recovery using Impella in a patient with cardiogenic shock.

## Introduction

Cardiogenic shock describes a life-threatening circulatory failure resulting in high rates of mortality [1]. The major mechanical circulatory support (MCS) used for cardiogenic shock include extracorporeal membrane oxygenation (ECMO), intra-aortic balloon pumping (IABP), and Impella. Long-term use of each MCS leads to serious complications such as infection, thrombosis, and bleeding. Therefore, the establishment of a strategy for rapid heart recovery in patients with cardiogenic shock is required. Impella is a percutaneous left ventricular assist device (LVAD) that maintains hemodynamic stability and also causes left ventricular (LV) mechanical unloading [2]. We present a comprehensive strategy for rapid heart recovery using Impella in patients with cardiogenic shock.

## Case presentation

A 57-year-old man with no specific medical history was admitted to the referral hospital with an episode of acute decompensated heart failure (HF). At the time of admission, his blood pressure (BP) was 94/74 mmHg, and his heart rate (HR) was 108 bpm. Echocardiography revealed severe LV dysfunction, with a left ventricular ejection fraction (LVEF) of 14%. Serum brain natriuretic peptide (BNP) level was markedly elevated (1164 pg/ml). Dobutamine and dopamine were administered to treat HF with hypotension and tissue hypoperfusion. Coronary angiography revealed no significant stenosis. Right ventricular endomyocardial biopsy showed nonspecific myocardial damage without inflammatory cellular infiltration. Therefore, the possibility of myocarditis was ruled out, and the patient was finally diagnosed with dilated cardiomyopathy. Dapagliflozin and low-dose candesartan were initiated for the recovery of cardiac function but were discontinued due to hypotension. After diuresis with furosemide and tolvaptan, hypoperfusion worsened, and the presence of acute kidney injury was revealed. Therefore, noradrenaline and IABP were initiated. He was sent to our hospital as a candidate for heart transplantation and an implantable LVAD.

**Figure 1:**
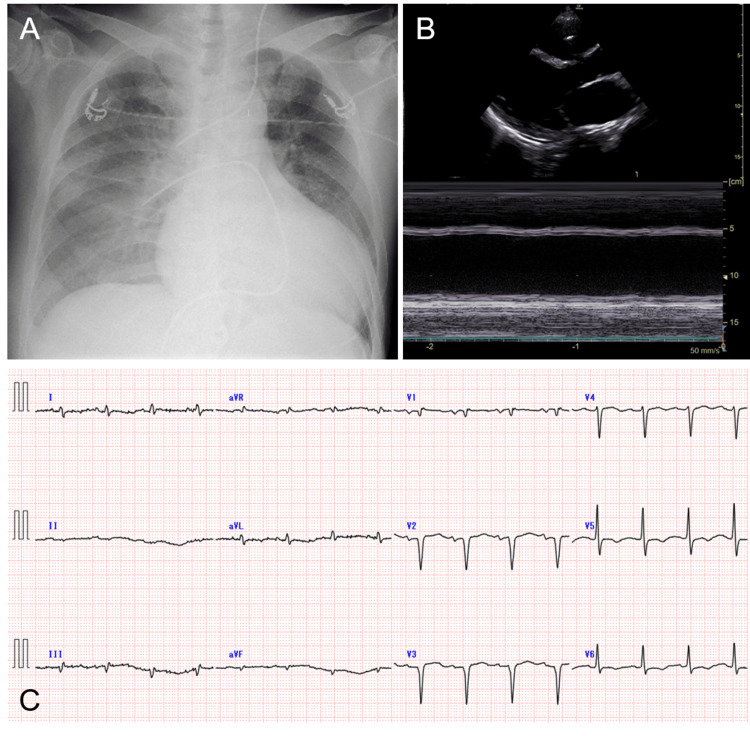
Chest radiography (A), echocardiography (B), and 12-lead electrocardiography (C) on admission

On arrival at our hospital, the patient’s BP was 109/54 mmHg, and HR was 93 bpm under IABP and the three inotropic agents. Chest radiography revealed marked cardiac enlargement with severe pulmonary congestion (Figure 1A). Echocardiography revealed an LV end-diastolic diameter of 68 mm and severe diffuse LV dysfunction (LVEF 10-15%) with moderate functional mitral regurgitation (Figure 1B). Electrocardiography showed a sinus rhythm with a heart rate of 94 bpm (Figure 1C).

**Figure 2:**
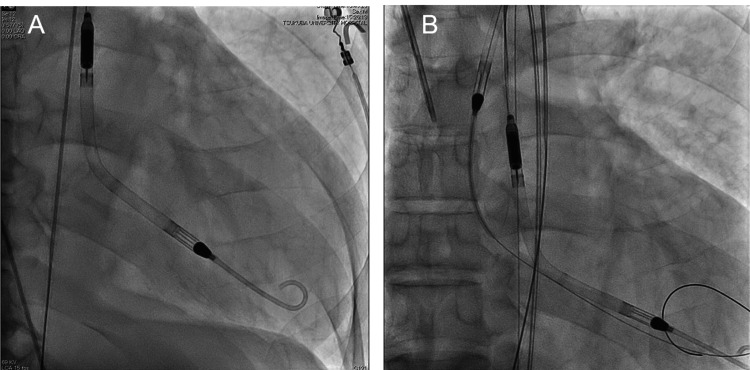
Insertion of first Impella CP (A) and exchange of Impella CP (B)

We initiated treatment with 25 mg/day eplerenone, 10 mg/day dapagliflozin, and 1.25 mg/day enalapril, with the support of IABP and inotropic agents. However, mixed venous oxygen saturation (SvO2) gradually decreased (minimum, 39%), and organ hypoperfusion worsened. No recovery of cardiac function was observed, and withdrawal from IABP or inotropic agents was considered impossible. The right heart catheter (RHC) showed severely low output (cardiac index: 1.8 L/min/m2, mean pulmonary capillary wedge pressure: 14 mmHg, and mean right atrial pressure: 11 mmHg). The pulmonary artery pulsatility index was 1.0 and relatively preserved. Therefore, an Impella CP was inserted via the right femoral artery, and the IABP was removed (Figure 2A). The Impella was set to a P8 drive (estimated blood flow 3.6 L/min). Initially, the opening of the aortic valve was not observed, and the mean arterial pressure was 75 mmHg. SvO2 recovered to 60-70%, and inotropic agents were tapered. Since the HR was approximately 90 bpm, 5 mg/day of ivabradine was initiated to reduce the HR and myocardial oxygen consumption. Furthermore, eplerenone, dapagliflozin, and enalapril were continued, but hemodynamics remained stable, unlike under IABP support. Because oxygenation was maintained, we managed the patient while awake without mechanical ventilation. On the 11th day after the start of the first Impella CP, we replaced the device, considering its durability. The second Impella CP was inserted through the left femoral artery, and the first one was removed. To avoid hemodynamic deterioration during the exchange, the second Impella was placed on standby in the ascending aorta and immediately inserted into the left ventricle as the first Impella was pulled out (Figure 2B).

**Figure 3:**
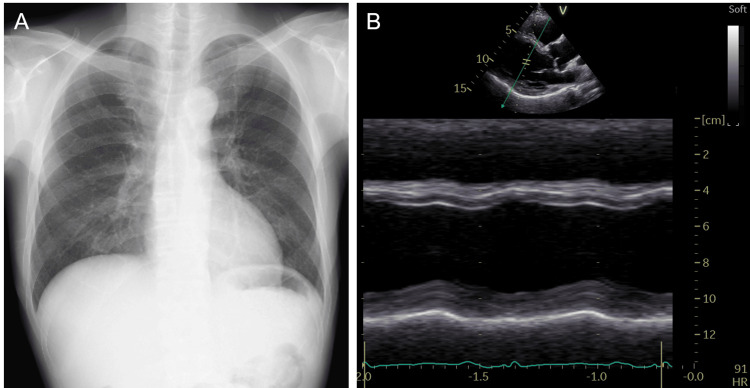
Chest radiography (A) and echocardiography (B) at discharge

To further reduce myocardial oxygen consumption and recover cardiac function, carvedilol (2.5 mg) was initiated and the dose of ivabradine was increased to 10 mg/day. Although severe cardiac dysfunction remained, the hemodynamics was maintained (BP 99/68 mmHg, HR 72 bpm, pulmonary capillary wedge pressure 6 mmHg, cardiac Index 3.2 L/min/m2, SvO2 69%) with partial support (P2). The patient was weaned from Impella 13 days after the start of the second Impella CP (total days of Impella support: 24 days). Echocardiography performed immediately after withdrawal revealed an LV end-diastolic diameter of 58 mm and an LVEF of 23%. His BP was 111/65 mmHg and his HR was 62/bpm. We switched enalapril to angiotensin receptor-neprilysin inhibitors (ARNI) and introduced vericiguat. One month after weaning from Impella, the patient could withdraw from inotropic agents. Chest radiography revealed improvement in cardiac enlargement, and LVEF improved to 40% (Figure 3A and 3B). The patient was eventually discharged without worsening HF. After 6 months, the serum BNP level normalized (13.0 pg/mL), and LVEF improved to 56%.

## Discussion

**Figure 4:**
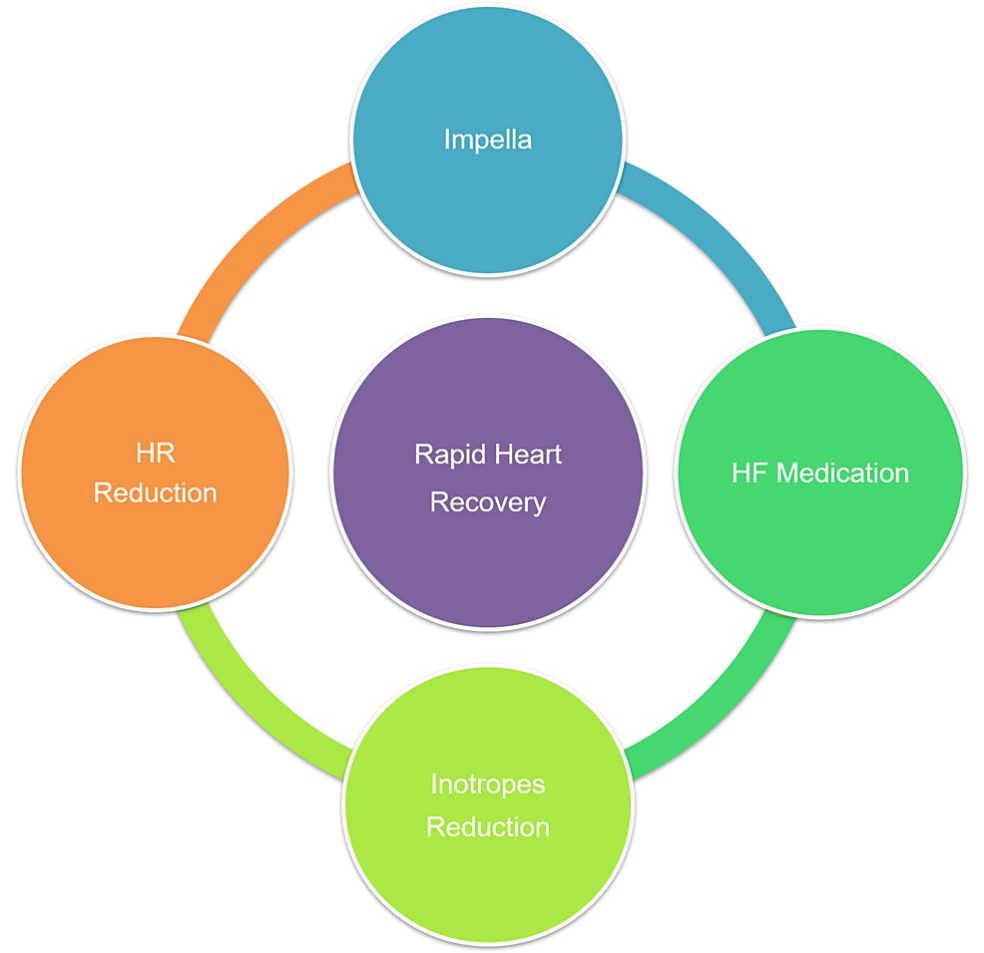
Comprehensive unloading strategy for rapid heart recovery Image Credits: Masayoshi Yamamoto This strategy consisted of (1) mechanical unloading and hemodynamic stability with Impella, (2) reduction of inotropic agents, (3) reduction of HR with ivabradine or β-blockers, and (4) initiation of standard HF medications. HR: heart rate, HF: heart failure.

We present a case of cardiogenic shock in which a comprehensive approach to unloading led to rapid recovery of cardiac function. ECMO, IABP, and Impella are the main MCSs used in patients with cardiogenic shock. Among these, Impella, a percutaneous LVAD, is the most potent MCS for LV mechanical unloading, depending on the assist flow. Long-term use of each MCS is challenging due to complications such as infection, thrombosis, bleeding, and device durability. However, since it takes time to sufficiently recover cardiac function and to be weaned off MCS, intensive treatment for withdrawal is required when MCS is initiated. In this case, we employed an intensive unloading strategy to achieve rapid heart recovery. This strategy consisted of (1) LV mechanical unloading and hemodynamic stability with Impella, (2) reduction of inotropic agents, (3) HR reduction with ivabradine or β-blockers, and (4) initiation of standard HF medications including sodium-glucose cotransporter 2 (SGLT2) inhibitors, mineralocorticoid receptor antagonists (MRA), and renin-angiotensin system (RAS) inhibitors from the acute phase (Figure 4).

IABP was initially used, but RAS and SGLT2 inhibitors could not be continued due to worsening hypotension and tissue hypoperfusion. However, ACE inhibitors, SGLT2 inhibitors, and MRA could be maintained without hemodynamic deterioration under Impella CP. "The fantastic four" are recommended for HF with reduced ejection fraction [3,4]. MRA, SGLT2Is, and ARNI have diuretic effects and may be effective in decongesting during the acute phase of HF. Recent studies have shown the efficacy and tolerance of ARNI and SGLT2 inhibitors when initiated in patients hospitalized for acute HF [5,6]. If BP and organ perfusion are maintained by Impella, these agents may contribute to decongestion, afterload reduction, and rapid recovery of cardiac function in patients with cardiogenic shock. A prior study reported that the cardiac index and mean arterial pressure increases were significantly greater in patients with Impella than in those with IABP [7]. Due to these characteristics, Impella is considered superior to IABP in terms of LV unloading and tolerability of HF medication in the acute phase.

Ivabradine inhibits the If channel in the sinoatrial node and lowers HR without affecting other aspects of cardiac function [8]. In the acute phase of HF, tachycardia is often accompanied by sympathetic nervous system activation as a compensatory mechanism. Tachycardia increases myocardial oxygen consumption and hinders the recovery of cardiac function. However, suppressing HR during the decompensated phase of HF may exacerbate the condition. Other major drugs that suppress HR include β-blockers. The distinction between ivabradine and β-blockers is that ivabradine has no negative inotropic effects. Therefore, compared to β-blockers, ivabradine carries a lower risk of exacerbating HF and can be initiated earlier in acute HF. Researchers in a case series of patients with cardiogenic shock reported that ivabradine was safely used to reduce HR in patients previously intolerant to β-blockade [9]. Furthermore, a significant reduction in HR after ivabradine administration in patients on VA-ECMO has been associated with native ventricular stroke volume improvement, allowing for a reduction in extracorporeal flow support and vasopressor administration [10]. In this case, because Impella can compensate for the reduction in intrinsic cardiac output after ivabradine administration, ivabradine could be initiated from the acute phase for LV unloading to facilitate HF recovery.

Points to consider in this strategy include that the use of Impella alone may necessitate the concurrent use of VA-ECMO or inotropic agents to support the right heart in patients with right heart dysfunction. Additionally, due to the risk of hypoperfusion resulting from a reduction in BP and HR, drug titration should be monitored using SvO2 or lactate to ensure that organ perfusion is maintained. Third, because oxygenation was preserved, we managed the patient while awake, without mechanical ventilation. Non-sedated management can prevent the risk of ventilator-associated pneumonia and allow for early detection if a stroke occurs. However, it can also cause physical and emotional distress, potentially increasing myocardial oxygen consumption. Depending on the patient's stress level, the use of a ventilator or sedation may need to be considered. Lastly, hypotension associated with HF medication may complicate withdrawal from MCS. In this instance, a change to Impella CP, which has a recommended tolerance of eight days, was necessary for further recovery of cardiac function. Recently, Impella 5.5, which allows for a longer period of support (30 days), has become available. This device may further enhance the utility of this comprehensive unloading strategy.

## Conclusions

We successfully weaned the patient from MCS and achieved a favorable outcome using the intensive unloading approach with Impella in a patient who was dependent on IABP and multiple inotropic agents. We believe that this report will contribute to the management of patients with cardiogenic shock. However, further evidence is needed to establish this approach.
